# Editorial: Molecular neuroscience: current and future challenges

**DOI:** 10.3389/fnmol.2026.2025299

**Published:** 2026-10-29

**Authors:** Jochen C. Meier, Detlev Boison, Clive R. Bramham, Gregg E. Homanics, Jean-Marc Taymans, Robert J. Vandenberg, Robert J. Harvey

**Affiliations:** 1 Institute for Cell- and Neurobiology, Division Cell Physiology, Technical University Braunschweig, Braunschweig, Germany; 2 Department of Neurosurgery, Robert Wood Johnson Medical School, Rutgers University, Piscataway, NJ, United States; 3 Brain Health Institute, Rutgers University, Piscataway, NJ, United States; 4 Rutgers Cancer Institute, New Brunswick, NJ, United States; 5 Department of Biomedicine, Mohn Research Center for the Brain, University of Bergen, Bergen, Norway; 6 Department of Anesthesiology and Perioperative Medicine, University of Pittsburgh School of Medicine, Pittsburgh, PA, United States; 7 Department of Neurobiology, University of Pittsburgh School of Medicine, Pittsburgh, PA, United States; 8 Department of Pharmacology and Chemical Biology, University of Pittsburgh School of Medicine, Pittsburgh, PA, United States; 9 Univ. Lille, Inserm, CHU Lille, UMR-S 1172-LilNCog-Lille Neuroscience and Cognition, Lille, France; 10 Molecular and Cellular Biomedicine, School of Medical Sciences, University of Sydney, Sydney, NSW, Australia; 11 School of Health, University of the Sunshine Coast, Maroochydore, QLD, Australia; 12 National PTSD Research Center, UniSC Thompson Institute, Birtinya, QLD, Australia

**Keywords:** activating transcription factor 4, age-related hearing loss, fragile X syndrome, glycine receptor autoantibodies, microexons, Parkinson's disease, sepsis-associated encephalopathy, synaptic plasticity

Molecular neuroscience has undergone a remarkable transformation over recent decades. The challenge is no longer simply to identify the neurotransmitters and associated molecular mechanisms that enable neuronal communication. In recent years, our focus has shifted toward understanding how molecular processes interact across spatial and temporal scales to shape cellular function, synaptic connectivity, neural circuits and whole organism behavior. Today, molecular neuroscience has advanced significantly, thanks in large part to new technologies like CRISPR gene editing, molecular and pharmacological probes, spatiotemporal gene expression control, optogenetics, induced pluripotent stem cell technology, advanced microscopy and single-cell RNA sequencing. These tools have enabled scientists to investigate differences in the molecular machinery of neurons and glia in unprecedented detail. However, understanding how these differences contribute to distinct cellular functions and network behaviors in health and disease states remains a daunting task. The Research Topic *Molecular Neuroscience: Current and Future Challenges* brings together studies that highlight recent advancements in Molecular Neuroscience, ranging from fundamental mechanisms of neuronal and synaptic function to cutting edge studies of neurodevelopmental/neurodegenerative disorders and newly-emerging therapeutic strategies.

A recurring theme across the collection is the importance of understanding molecular mechanisms in a given cellular and functional context. For example, Chojnacka et al. investigated auditory hypersensitivity in fragile X syndrome (FXS), demonstrating delayed structural maturation of inner hair cell ribbon synapses in *Fmr1* knockout mice. Although mature synapses lacked major ultrastructural abnormalities, altered postnatal synaptic development provided a potential link between loss of FMRP and the auditory abnormalities associated with FXS. At another level of synaptic organization, Gobbo et al. examined the relationship between neuronal activation and synaptic plasticity during hippocampal memory formation. Using molecular reporters of neuronal activation and postsynaptic potentiation, they demonstrated that the relative weight and spatial distribution of potentiated dendritic spines in hippocampal CA1 neurons differ between memory encoding and retrieval. Together, these studies illustrate how molecular changes at individual synapses can potentially influence sensory processing and higher-order functions such as memory.

The importance of ion channels and neurotransmitter receptors is similarly evident. Deng et al. reviewed the contribution of transient receptor potential (TRP) channels to intracellular calcium dysregulation and neuronal hyperexcitability in epilepsy. The diversity of TRP channel subtypes and their interactions with intracellular calcium, oxidative stress and other signaling pathways illustrates both the therapeutic opportunities and complexities associated with targeting neuronal ion channels in disease states. Wiessler et al. investigated autoimmune neurological disease caused by autoantibodies directed against inhibitory glycine receptors (GlyRs), key regulators of synaptic inhibition in the spinal cord and brainstem. Their findings indicated considerable patient-specific heterogeneity in GlyR subunit autoantibody recognition, with some antibodies recognizing epitopes in multiple subunits (α1-3 and β), complicating the identification of a universal neutralizing epitope. This work highlights an important challenge for precision medicine: even when a molecular target is known, individual differences in the mechanisms affecting that target may necessitate personalized therapeutic approaches.

The collection also emphasizes that molecular function cannot necessarily be inferred from gene or protein expression alone. Pronina et al. examined tyrosine hydroxylase (TH) expressed in neurons of the hypothalamic supraoptic nucleus (SN). Despite detecting TH expression, their findings indicate that the TH protein in the SN does not exhibit the expected enzymatic activity required for L-DOPA synthesis, potentially because of potential differences in the N-terminal regulatory region. This study provides a useful reminder that the functional significance of a molecular phenotype requires investigation beyond simple transcript or protein analysis.

Gene regulation and cellular stress responses provide another level of complexity. For example, the alternative use of microexons, short exonic sequences of 3–30 nucleotides that undergo alternative splicing, has been implicated in several neurodevelopmental disorders. Mehnert et al. investigated microexon-associated junction usage in Parkinson's disease (PD) and spinocerebellar ataxia type 3 using deep RNA-sequencing of human postmortem brain tissue. Although no disease-associated events survived correction for multiple testing, substantially more microexons were detectable in human brain tissue than in corresponding cellular models. These findings emphasize both the subtlety of post-transcriptional regulation and the limitations inherent in extrapolating from simplified cellular systems to the complexity of human brain tissue. Huang et al. reviewed the role of activating transcription factor 4 (ATF4), a central component of the neuronal integrated stress response (ISR). They highlighted the context-dependent nature of ATF4 signaling, which can support neuronal homeostasis under some circumstances while contributing to neuroinflammation, ferroptosis and neuronal dysfunction when chronically dysregulated. Consequently, therapeutic intervention may require modulation of the timing and magnitude of stress signaling rather than simple pathway inhibition.

The importance of interactions beyond neurons themselves is particularly apparent in Parkinson's disease. Kroker Kimber and Tremblay reviewed communication between the gut microbiome and microglia, highlighting evidence that microbial dysbiosis may influence systemic inflammation, microglial activation and α-synuclein pathology. Probiotic interventions represent an intriguing approach to modifying this gut-brain communication, although direct evidence for modulation of human brain microglia remains limited. Tumpa et al. extended this perspective by considering interactions between peripheral and central innate and adaptive immunity in Parkinson's disease. Together, these contributions illustrate an increasingly important concept in molecular neuroscience: brain disorders cannot always be understood by studying the brain in isolation.

This broader “‘systems”' perspective is also evident in two contributions examining neurological dysfunction from translational viewpoints. Wen et al. reviewed the multifactorial molecular basis of age-related hearing loss (ARHL), also known as or presbycusis, encompassing oxidative stress, inflammation, altered autophagy, cellular senescence, genetic susceptibility and environmental influences. Emerging approaches including gene therapy, regenerative strategies, targeted drug delivery and biomarker-based early intervention—which illustrate the potential for mechanistic discoveries to generate new therapeutic opportunities, while also emphasizing the substantial challenges of clinical translation. Yan et al. similarly integrated molecular pathophysiology with neuroimaging in a review of sepsis-associated encephalopathy, linking blood-brain barrier disruption, neuroinflammation, neurotransmitter imbalance and neuronal and glial injury with measurable imaging phenotypes. Such approaches provide an important bridge between molecular mechanisms and clinically accessible biomarkers.

Taken together, the contributions to this Research Topic illustrate both the extraordinary progress and the continuing challenges of molecular neuroscience. Increasingly powerful experimental approaches allow investigation from gene regulation and protein function through individual synapses, neural circuits and interactions between the nervous, immune and peripheral systems. However, greater resolution has also revealed additional biological heterogeneity and complexity. Future progress will depend not only on identifying new molecular mechanisms, but on determining when, where and in which cells these mechanisms operate, how they interact across body organs and systems, and how faithfully our cellular or animal models represent human disease. Ultimately, meeting these challenges will be essential if advances in molecular neuroscience are to be translated into meaningful biomarkers, precision interventions and effective therapies for disorders of the central and peripheral nervous system.

